# Casting a long shadow: Exploring the link between childhood maltreatment and cancer in adulthood

**DOI:** 10.1371/journal.pone.0345411

**Published:** 2026-04-22

**Authors:** Matthew Robert Langiano, Carmine Malfitano, Esme Fuller-Thomson

**Affiliations:** 1 Factor-Inwentash Faculty of Social Work, University of Toronto, Toronto, Ontario, Canada; 2 Princess Margaret Cancer Centre, University Health Network, Toronto, Ontario, Canada; 3 Centre for Psychology and Emotional Health, Toronto, Ontario, Canada; 4 Institute for Life Course and Aging, University of Toronto, Toronto, Ontario, Canada; Public Library of Science, UNITED KINGDOM OF GREAT BRITAIN AND NORTHERN IRELAND

## Abstract

Childhood adversities, including physical abuse, sexual abuse, and exposure to parental domestic violence (PDV), have been linked to negative health outcomes in adulthood, yet their specific association with cancer in older Canadians remains understudied. The current study investigated the associations between these specific childhood adversities and cancer in Canadian older adults. We conducted a secondary analysis of the nationally representative cross-sectional 2022 Canadian Mental Health and Access to Care Survey (MHACS). The sample size was 2,636 Canadians aged 65 and older. Bivariate and logistic regression models assessed the relationship between self-reported cancer diagnoses and self-reported childhood adversities – childhood sexual abuse (CSA) (including unwanted touch/fondling and Childhood Sexual Violence with Coercion (CSVC)), childhood physical abuse (CPA), and PDV. Logistic regression analyses controlled for demographic characteristics, socioeconomic status, health behaviors, chronic pain, mental health conditions, and psychosocial factors. In the bivariate analyses, those who had experienced childhood adversities had a significantly (p < 0.05) higher prevalence of cancer than those who had not (CPA 28% vs. 20.7%; PDV 26.5% vs. 20.6%; CSVC 35.5%; unwanted touch/fondling 25.5%, no CSA 20%). After comprehensive adjustment for demographic, socioeconomic, behavioral, and psychosocial factors, CSVC was associated with double the odds of cancer diagnosis (odds ratio = 2.05; 95% confidence interval 1.41–2.98), but the other forms of childhood adversity no longer reached statistical significance. Childhood adversities are associated with an elevated prevalence of cancer in later life, and the association between CSVC and cancer remained significant even after adjustments for most of the known risk factors for cancer. Further longitudinal research exploring stress-induced biological changes and immune dysregulation is needed among those with a history of childhood adversities. These findings lend support to the integration of trauma-informed care into oncology services.

## Introduction

Cancer is a significant global health challenge, with close to 20 million new cases and approximately 9.7 million deaths recorded in 2022 [1]. Cancer incidence rates begin to rise in midlife and peak in older adulthood [2]. Despite substantial advancements in early detection and treatment, cancer continues to impose a significant burden on individuals, families, and healthcare systems. Cancer remains as one of the leading causes of death, accounting for a substantial proportion of all mortality nationwide [3].

Tobacco use, diet, and environmental exposures are well-established risk factors for a wide range of later-life health conditions, including cancer [4]. Emerging research suggests that psychosocial determinants, including adverse childhood experiences (ACEs) may also be associated with many chronic health conditions and negative health behaviors across the lifespan [5–7], potentially increasing susceptibility to cancer [8].

Some studies from the United States (US), Great Britain, Canada, Finland, and Saudi Arabia link physical abuse to long-term health consequences, including an elevated risk of cancer [9], and further research suggests that sexual abuse also increases cancer risk [10–12]. In addition, two US studies found that individuals exposed to parental intimate partner violence had significantly higher odds of developing cancer in adulthood [13,14].

However, the association between specific types of childhood adversity, particularly childhood sexual abuse (CSA), childhood physical abuse (CPA), and parental domestic violence (PDV), and cancer risk in adulthood remains understudied in Canadian samples (for important exceptions, please see [12,15]). While Hovdestad et al. [12] made a significant contribution by identifying a robust association between childhood maltreatment and cancer among Canadian adult population, their analyses did not focus solely on older Canadians and did not adjust for several important potential cancer risk factors. Notably, their models did not include covariates such as disabling chronic pain, the number of non-cancer chronic health conditions, social support, spiritual coping, or substance use, variables that may confound the relationship between childhood adversities and cancer risk. These variables are important because childhood adversity can influence lifelong coping, stress physiology, and health behaviors. Adaptivecoping strategies, such as social support or spiritual coping, may buffer stress-related effects, whereas maladaptive coping strategies, including substance use, is more common after childhood trauma and is linked to poorer health. Accounting for these factors strengthens the ability to isolate the association of childhood adversity with cancer.

Sex, race, and immigrant status were also incorporated as covariates because these demographic factors shape both the likelihood of experiencing childhood adversity and broader health trajectories in adulthood. Trauma exposure patterns may differ by sex and across racial or Indigenous groups, and immigrant populations may face distinct forms of life course adversity. Including these variables helps ensure that observed associations are not driven by demographic differences.

In order to explore the direct association between childhood adversities and cancer, it is essential to take into account factors which may indirectly increase cancer susceptibility through intermediary psychosocial factors. These risk factors for cancer include reduced social support [16], lower socioeconomic status (SES) in adulthood [17], and chronic pain [18]. It is also important to evaluate the potential role of health behaviors. Bochicchio et al. [19] noted that the evidence linking ACEs to health risk behaviors is somewhat mixed; while ACEs are associated with behaviors such as overeating and smoking among sexual minority women, other studies have found no such associations. A Canadian study found that child maltreatment was associated with smoking [20]. However, Lee et al. [21], using a United States (US) based national sample of sexual and gender minority adults aged 18 and older, did not find evidence of an association between ACEs and smoking within this population.

Nationally representative data from Canada indicate that approximately 32% of individuals report experiencing CPA, 14% report CSA, and 18% report exposure to PDV [22]. Further, Hovdestad et al. [12] found that among women, 8.1% reported severe CSA (including those who experienced it one or more times), and 6.4% reported less severe forms, such as unwanted sexual touching.

Given the complexity of cancer etiology, and the magnitude of ACEs, understanding if early-life adversities are associated with cancer in a nationally representative sample of older Canadians is critical to informing prevention efforts and public health interventions. The current paper uses nationally representative data on older Canadians from the 2022 Canadian Mental Health and Access to Care Survey Data (MHACS) to 1) examine whether CSA, CPA, and exposure to PDV are associated with cancer in late adulthood and 2) to determine if the relationship between these ACEs and cancer persist after accounting for potential confounders, including sex, race, immigrant status, marital status, income, education, physical activity, smoking history, history of drug and/or alcohol abuse, social support, disabling chronic pain, number of chronic health conditions (excluding cancer), and use of spiritual coping.

Based on prior literature, we hypothesized that older adults with histories of CSA, CPA, and/or exposure to PDV would have a higher prevalence and odds of reporting a cancer diagnosis compared to those without these adversities. We further hypothesized that these associations would be attenuated after adjustment for demographic, socioeconomic, behavioral, and psychosocial factors.

## Methods

We built on our previous studies which examined the link between ACEs and health outcomes in the 2012 Canadian Community Health Survey-Mental Health (CCHS-MH) (see [23]), through the use of data from the 2022 MHACS for the current study [24]. As has been described elsewhere [23], using a stratified simple random sample and cross-sectional design, this survey is representative of community-dwelling Canadians aged 15 and older living in the 10 provinces of Canada and covers 97.5% of the target population [25]. Our analyses were limited to MHACS respondents aged 65 and older in order to focus on the age group with the most elevated prevalence of a lifetime cancer diagnosis and to enhance the temporal clarity between exposure and outcome. The survey response rate for those aged 65 and older was 30.4% [25]. There were some missing data on many of the variables included in the logistic regression. If the missing data for the variable was less than 1% of the sample of older adults, those in the missing category were excluded from the analysis. If the prevalence of missing respondents for a variable was above 1% of the sample, a missing category was created for that variable, and was included in the analyses. The current analysis included the subsample of those aged 65 and older with complete data on ACEs and cancer, as well as each of the independent measures included in the analysis, as described below. This resulted in a loss of 59 respondents, which represents 2.2% of those aged 65 and older. Consistent with other similar studies [23,26], we used a constant sample size for all the analyses to facilitate direct comparisons across models. Our final sample consisted of 2,636 Canadians aged 65 and older. As the analysis was conducted using publicly available, de-identified data, the study was exempt from Institutional Review Board (IRB) oversight.

### Measures

#### Key exposure variables.

The MHACS questionnaire [27] contains a section on childhood adversities which begins with the statement “the next few questions are about things that may have happened to you before you were 16 in your school, in your neighbourhood, or in your family.” Individuals were coded as having been exposed to PDV if they reported they had seen or heard at least one time their “parents, step-parents or guardians hit each other or another adult” aged 18 or over in their home. CSA was assessed by two questions: a) “Before age 16, how many times did an adult touch you against your will in any sexual way? By this, I mean anything from unwanted touching or grabbing to kissing or fondling,” and b) “How many times did an adult force you or attempt to force you into any unwanted sexual activity, by threatening you, holding you down, or hurting you in some way?”If an individual responded ‘at least one time’ to question b, they were categorized as having experienced Childhood Sexual Violence Involving Threat, Physical Coercion or Physical Harm (hereafter Childhood Sexual Violence with Coercion; CSVC).If they responded ‘at least one time’ to question a but responded ‘never’ to question b, they were categorized as having experienced ‘unwanted touch/fondling’. If they responded ‘never’ to both questions a and b, they were categorized as never having experienced CSA. Individuals were coded as having experienced CPA if they reported that an adult had kicked, bit, punch, choked, burned, or physically attacked them at least once.

#### Outcome variables.

The MHACS contains a section of questions on chronic diseases, which begins with the preamble “Now I’d like to ask about certain long-term health conditions which you may have….We are interested in ‘long-term conditions’ which are expected to last or have already lasted 6 months or more and that have been diagnosed by a health professional.” The respondents were asked 1) “Do you have cancer” and 2) “Have you ever been diagnosed with cancer?” Respondents who answered yes to either 1 or 2 were classified as having cancer and those who responded no to both 1 and 2 were classified as not having cancer.

#### Control variables.

The following control variables were included in the analysis: self-reported sex at birth (male versus female), race/ethnicity based upon self-report [White (includes Indigenous respondents) versus Visible Minority], immigrant (immigrant versus Canadian-born), marital status (married/common-law vs widowed/divorced/never married), income (<$20,000, $20,000-$39,999, $40,000-$59,999, $60,000-$79,999, $80,000-$99,999, $100,000-$149,999, $150,000 or more), education level was measured by the highest level of achieved education (less than high school, high school graduate or equivalent, some post-secondary school, bachelor’s degree, post-bachelor’s degree, missing data), physical activity (whether or not the respondent participated in any moderate or vigorous physical activity in the 7 days preceding the survey), smoking history (never smoker, former smoker, current smoker). Two forms of substance use disorders were also included based on the World Health Organization-Composite International Diagnostic Interview (WHO-CIDI) [28]: “Lifetime Drug Abuse or Dependence (including cannabis)” and “Lifetime Alcohol Abuse or Dependence.” Spiritual coping was based upon the question “In general, how important are religious or spiritual beliefs in your daily life?” (very important, somewhat or not very important, not important at all, missing data). Social support was based upon two questions: 1) “how much you agree … you can count on people that you know to help you deal with your biggest stress” (strongly agree, neither agree nor disagree, disagree/strong disagree, missing data), and 2) “in the past month, how often did you feel ... that you had warm and trusting relationships with others?” (daily, almost daily, from never to 2–3 times per week, missing data). *Chronic Pain* was assessed through two questions in a section of the survey on pain and discomfort beginning with the statement: “The next set of questions asks about the level of pain or discomfort you usually experience. They are not about illnesses like colds that affect people for short periods of time.” Respondents were determined as being regularly in pain if they responded “no” to the question “Are you usually free of pain or discomfort?” If they responded no, they were asked “How many activities does your pain or discomfort prevent?” The response categories of the combined variable were (no pain or no activities prevented by pain versus pain prevents few/some/most activities, versus missing data). The number of self-reported chronic health conditions (excluding cancer) were categorized as none, 1, 2, 3 or more chronic conditions, and a missing data category was included.

### Statistical analyses

All analyses were undertaken using SPSS v. 29. Univariate and bivariate statistics (i.e., frequency/percentages and chi-square tests) are presented to describe the sample and the relationships between each of the study variables and cancer. To address our research questions, two binary logistic regression analyses with cancer as the outcome were conducted, the first included all types of childhood adversities, the second included all types of childhood adversities and all the control variables (described above). Odds ratios (ORs) with 95% confidence intervals (CIs) were calculated by exponentiating the logistic regression β-coefficients. To assess model fit, Nagelkerke Pseudo R² values were reported, reflecting the percentage of variance explained. Data from all the analyses were weighted to adjust for the probability of selection and non-response. Sample sizes are reported in their unweighted form. Statistical significance was defined as p < 0.05.

## Results

A description of the overall sample of Canadians aged 65 and older is presented in Table 1, column 2. One in five (21.1%) reported that they had been diagnosed with cancer at some point in their life. With respect to the ACEs, 4.4% experienced unwanted touch/fondling, 5.9% experienced CSVC, 7.8% experienced CPA, and 13.2% experienced PDV. The majority of respondents identified as female (53.1%), White or Indigenous (83.4%), Canadian-born (68.6%), married or common-law (64.4%), with a household income above $60,000 (61.8%), more than a high school education (56.9%), physically active (71.1%), without a history of drug use disorder (90.7%) or alcohol use disorder (81.7%). The majority also did not rate their religious or spiritual beliefs in your daily life as ‘very important’ (62.2%), reported they could count on people to help them deal with their biggest stressor (79%), were free of disabling chronic pain (77.8%) and had one or fewer chronic conditions (excluding cancer) (60.8%).

**Table 1:** Total Prevalence of Sociodemographic Characteristics & Health Behaviors and Prevalence of Cancer by These Characteristics Among Canadians Aged 65 and older (N = 2,636).

Characteristics	Total % (Column adds to 100%)	% with cancer	P-value
**Cancer**			
Does not have cancer	78.9	N/A	
Had a cancer diagnosis	21.1	N/A	
**Sexual Abuse**			**<0.001**
No Sexual Abuse	86.8	20	
Unwanted touch/fondling	4.4	25.5	
Childhood Sexual Violence with Coercion (CSVC)	5.9	35.5	
Missing data on sexual abuse (Refused/Don’t know)	3	17.9	
**Domestic Violence**			**0.003**
No Domestic Violence	83.5	20.6	
Domestic violence experienced once or more	13.2	26.5	
Missing data (Refused/Don’t know)	3.3	11.1	
**Adult Kick, Bite, Punch, Choke, Burn or Physically Attack at Least Once**			**0.013**
No adult kick, bite, punch, choke, burn or physically attack	89.4	20.7	
Adult kick, bite, punch, choke, burn or physically attack at least once	7.8	28	
Missing data (Refused/ Don't know)	2.8	13.9	
**Sex**			0.28
Male	46.9	20.2	
Female	53.1	21.8	
**Race**			**0.001**
Visible Minority	13.9	13.7	
White	83.4	22.3	
Missing Data	2.7	19.1	
**Immigrant Status**			**0.01**
Canadian Born	68.6	22.5	
Immigrant	29.8	18.1	
Missing Data	1.6	11.9	
**Marital Status**			0.47
Never Married/Widowed/Separated/Divorced	35.6	21.8	
Married/Common-Law	64.4	20.6	
**Income Level**			0.55
Less than $20,000 including no income	2.1	16	
$20,000–39,999	18.1	23.5	
$40,000–59,999	18	21	
$60,000–79,999	15.5	21	
$80,000–99,999	14	21.5	
$100,000–149,999	18.3	21.5	
$150,000 or more	14	17.7	
**Education Level**			0.20
Less than high school diploma or equivalent	14.8	22	
High school diploma or equivalency certificate	26.8	21.2	
Trades/College/University certificate below bachelor's level	33	19	
Bachelor’s degree	13.8	23.4	
University certificate/diploma/degree above bachelor’s level	10.1	24.2	
Missing Data	1.6	12.1	
**Moderate Activity Within 7 Days**			0.27
No	27.5	23.1	
Yes	71.1	20.2	
Missing Data	1.5	23.8	
**Smoking History**			**0.04**
Never Smoked	37.2	19.8	
Former Smoker	52.7	22.8	
Current Smoker	10.1	16.7	
**Drug Use Disorder**			0.75
No drug abuse or dependence	90.7	20.9	
Drug abuse or dependence during lifetime	3.8	22.4	
Missing Data	5.5	23	
**Alcohol Use Disorder**			**<0.001**
No alcohol abuse or dependence	81.7	19.8	
Alcohol abuse or dependence during lifetime	15.2	24.7	
Missing Data	3.2	36.4	
**Spirituality Level**			0.59
Very important	35.6	20.1	
Somewhat or not very important	46.1	22.2	
Not important at all	16.1	19.8	
Missing data	2.2	19.9	
**Can Count On People You Know To Deal With The Biggest Stress**			**0.04**
Strongly agree	79	21.8	
Neither agree nor disagree	7.9	17.4	
Disagree/Strongly Disagree	7	22.9	
Missing data	6.1	13.7	
**Frequency of Warm and Trusting Relationship With Others During the Past Month**			0.81
Daily	57.6	21.3	
Almost daily	26.8	19.8	
2-3 times a week including never	12.7	22.1	
Missing data	2.9	22.5	
**Pain Preventing Activities**			**0.004**
No pain or no activities prevented by pain	77.8	19.6	
Pain prevents few/some/most activities	21.2	26.1	
Missing data	1	24.2	
**Chronic Conditions (not including cancer)**			**0.04**
No chronic condition	29.7	18.9	
1 chronic condition	31.1	19.3	
2 chronic conditions	20.5	24	
3 or more chronic conditions	15.3	23.4	
Missing data	3.5	27.4	

2022 Canadian Mental Health and Access to Care Survey Data (MHACS)

Bold indicates statistical significance with p < 0.05**.**

In Table 1, column 3, the bivariate analyses indicated that those who had experienced childhood adversities had a significantly (p < 0.05) higher prevalence of cancer than those who had not experienced these adversities (CPA 28% vs 20.7%; PDV 26.5% vs 20.6%; CSVC 35,5%; unwanted touching/fondling 25.5%, vs no CSA 20%). The prevalence of cancer was also significantly higher among White or Indigenous respondents compared to visible minority respondents (22.3% vs 13.7%), among Canadian born compared to immigrants (22.5% vs 18.1%), among former smokers (22.8%) in comparison to current smokers (16.7%) or never smokers (19.8%), among those with a history of alcohol use disorder vs those without (24.7% vs 19.8%), among those in disabling pain (26.1% vs 19.6%) and among those with more chronic conditions compared to those without.

After comprehensive adjustment for demographic, socioeconomic, behavioral, and psychosocial factors, CSVC was associated with double the odds of cancer (OR = 2.05; 95% CI 1.41–2.98) (Table 2). All other forms of abuse ranged from 1.21 to 1.25 higher odds of cancer, but they did not reach statistical significance (i.e., p > 0.05). The only other characteristic significantly associated with higher odds of cancer in the logistic regression analysis was White or Indigenous race/ethnicity compared to visible minority status.

## Discussion

### Childhood sexual abuse and cancer risk in older adulthood

The prevalence of cancer was significantly higher among respondents who experienced CSA compared to those without CSA (CSVC: 35.5%; Unwanted touch/fondling: 25.5%; no CSA: 20%). In the logistic regression analyses, after adjusting for demographic, socioeconomic, behavioral, and psychosocial covariates, CSVC remained significantly associated with approximately double the odds of cancer compared to individuals without CSA (OR = 2.05, 95% CI = 1.41–2.98). Unwanted touch/fondling, however, was not significantly associated with cancer risk after covariate adjustments.

The current study’s findings on older Canadians align with Hovdestad et al. [12], who found that CSA was significantly associated with increased cancer risk, particularly among adult Canadian women. Their study identified a dose-response relationship between CSA severity and cancer prevalence. While they used a tiered model based on frequency and severity, our dichotomous model focusing on CSVC still yielded a similarly robust association. This suggests that even a single experience of sexual violence involving coercion may be associated with long-lasting physiological consequences relevant to cancer risk, particularly in older adulthood.

Earlier research suggests that survivors of CSVC show greater long-term dysregulation of stress-response systems, particularly chronic alterations in cortisol production and heightened inflammation [29,30]. Such biological disruptions have been implicated in the development and progression of cancer through mechanisms involving chronic inflammation and immune dysregulation [9,31,32]. Thus, CSVC may exert independent physiological effects, increasing vulnerability to cancer in ways distinct from other forms of childhood adversity.

### Childhood Physical abuse, parental domestic violence, and cancer risk

Individuals who experienced CPA in the current study had a significantly higher prevalence of cancer compared to those who did not (28% vs. 20.7%). Similarly, respondents exposed to PDV had an elevated cancer prevalence relative to unexposed individuals (26.5% vs. 20.6%). In logistic regression analyses, however, after adjusting for all forms of childhood adversity, neither CPA nor PDV significantly predicted cancer risk. Further adjustment for demographic, socioeconomic, behavioral, and psychosocial covariates failed to further attenuate the odds.

Hovdestad et al's. study [12] had a much larger sample size than was available in the MHACS, which provided them with adequate statistical power to conduct sex-specific analyses. Their findings indicated that CPA and PDV were significantly associated with cancer for women, but not for men, in their 2012 nationally representative sample of adults. Among women, inclusion of a wide range of potential mediating factors (e.g., smoking, stress, depression and substance use disorders) provided moderate attenuation of the direct relationship with cancer. It is unclear why adjustment for other potential risk factors was associated with a much greater attenuation of the childhood adversity-cancer relationship in the 2012 sample of women than in our current 2022 mixed sex sample. It is important to note that they used a broader definition of CPA, including less severe behaviors like slapping and pushing, whereas our measure focused exclusively on more severe acts such as kicking or choking.

Hovdestad and colleague’s findings align with previous literature, indicating that the CPA and PDV association with cancer often diminish or become non-significant after adjusting for broader psychosocial and environmental factors [15]. One possible explanation for this attenuation is that CPA and PDV commonly co-occur within broader contexts of familial dysfunction, socioeconomic disadvantage, and chronic stress – which are associated with long-term health outcomes [33]. Furthermore, the initially observed associations between these adversities and cancer risk in bivariate analyses may reflect indirect pathways through shared risk factors – such as childhood poverty [34], and lifetime cumulative stress exposure [35] – rather than the direct effects of CPA or PDV themselves. Future research should explore how these cumulative exposures, alongside intervening stressors across the life course, influence cancer susceptibility, potentially through chronic physiological stress responses and prolonged inflammatory states.

### Potential mechanisms linking ACEs to cancer risk

Although this observational study prohibits identification of causal mechanisms, we hypothesize the association between childhood adversities— CSVC, unwanted touch/fondling, CPA, and PDV—and elevated cancer prevalence in later life may be partially explained by biological embedding, a process by which early-life stressors disrupt stress regulation and immune function, influencing long-term health [36]. ACEs such as CSVC, unwanted touch/fondling, CPA, and PDV, have been linked to dysregulation of the hypothalamic-pituitary-adrenal (HPA) axis, chronic inflammation, and immune suppression—all of which are implicated in cancer pathogenesis through mechanisms like oxidative stress, DNA damage, and impaired tumor surveillance [37–39].

There is evidence that childhood adversity may contribute to long-term physiological changes that could be relevant to cancer risk. Studies have shown that adults with histories of childhood maltreatment exhibit higher levels of systemic inflammation than those without such experiences [32]. A recent meta-analysis similarly found that childhood adversity is associated with elevated inflammatory activity across developmental stages [40]. Although our study cannot establish causality, these findings suggest that early stress may become biologically embedded over time, contributing to chronic low-grade inflammation implicated in the development of chronic diseases, including cancer [32].

While only CSVC emerged as a significant predictor of cancer after controlling for a wide range of factors, the ORs for CPA, PDV, and unwanted touch/fondling were similar in magnitude (approximately 20% higher odds than their peers without these early childhood adversities). This pattern suggests that common physiological disruptions may underlie all ACEs, even if statistical significance was not reached when all these adversities were considered simultaneously. Future research with larger studies are needed to explore this hypothesis, and studies should examine whether the cumulative impact or severity of adversity differentially affects biological pathways linked to cancer development.

### Unexpected findings & covariate adjustments

It was expected that the association between CSVC and cancer risk would be substantially attenuated after adjusting for health behaviors, SES, and psychosocial factors. When all three ACEs were modeled together in Block 1 (see Table 2) of our regression, only CSVC remained statistically significant. The addition of further covariates in Block 2 (See Table 2) did not substantially alter the ORs. This stability highlights the independent contribution of CSVC to cancer risk—an insight echoed by Hovdestad et al. [12], who similarly found that CSA (particularly among women) remained associated with cancer after accounting for co-occurring maltreatment and risk behaviors.

**Table 2:** Logistic Regression of Factors Associated with Lifetime Cancer Among Canadians Aged 65 and Older (N = 2,636).

	Analyses Adjusted for 3 Adverse Childhood Experiences Only (Block 1)	Fully Adjusted Analyses (Block 2)
VARIABLE NAMES	Odds Ratio (95% CI)	Odds Ratio (95% CI)
**Sexual Abuse**		
No Sexual Abuse	1.00 (Reference)	1.00 (Reference)
Unwanted touch/fondling	1.33 (0.87, 2.05)	1.25 (0.80, 1.95)
Childhood Sexual Violence with Coercion (CSVC)	**2.09 (1.46, 2.98)**	**2.05 (1.41, 2.98)**
Missing data on sexual abuse (Refused/Don’t know)	1.84 (0.78, 4.36)	1.46 (0.58, 3.66)
**Domestic Violence**		
No Domestic Violence	1.00 (Reference)	1.00 (Reference)
Domestic violence experienced once or more	1.19 (0.90, 1.57)	1.22 (0.92, 1.63)
Missing data (Refused/Don’t know)	**0.32 (0.11, 0.94)**	0.35 (0.12, 1.03)
**Adult Kick, Bite, Punch, Choke, Burn or Physically Attack at Least Once**		
No adult kick, bite, punch, choke, burn or physically attack	1.00 (Reference)	1.00 Reference
Adult kick, bite, punch, choke, burn or physically attack at least once	1.23 (0.87, 1.74)	1.21 (0.84, 1.73)
Missing data (Refused/ Don't know)	0.97 (0.33, 2.89)	1.12 (0.37, 3.37)
**Sex**		
Male		1.00 (Reference)
Female		1.09 (0.87, 1.35)
**Visible Minority, White or Missing data**		
Visible Minority		1.00 (Reference)
White		**1.61 (1.09, 2.38)**
Missing data (Refused/Don't know)		**2.44 (1.07, 5.57)**
**Immigrant Status**		
Canadian born		1.00 (Reference)
Immigrant		0.95 (0.74, 1.24)
Missing data (Refused/ Don't know)		0.58 (0.08, 3.97)
**Marital Status**		
Never Married/Widowed/Separated/Divorced		0.99 (0.79, 1.25)
Married/Common-Law		1.00 (Reference)
**Income Level**		
Less than $20,000 including no income		1.00 (Reference)
$20,000–39,999		0.94 (0.42, 2.10)
$40,000–59,999		1.37 (0.93, 2.03)
$60,000–79,999		1.27 (0.88, 1.84)
$80,000–99,999		1.18 (0.81, 1.73)
$100,000–149,999		1.14 (0.78, 1.67)
$150,000 or more		1.22 (0.86, 1.74)
**Education Level**		
Less than high school diploma or equivalent		1.00 (Reference)
High school diploma or equivalency certificate		1.02 (0.74, 1.39)
Trades/College/University certificate below bachelor's level		0.89 (0.65, 1.21)
Bachelor’s degree		1.28 (0.89, 1.86)
University certificate/diploma/degree above bachelor’s level		1.36 (0.91, 2.03)
Missing Data		0.51 (0.08, 3.12)
**Moderate Activity Within 7 Days**		
Yes		1.00 (Reference)
No		1.16 (0.93, 1.44)
Missing Data		0.93 (0.42, 2.08)
**Smoking History**		
Never Smoked		1.00 (Reference)
Former Smoker		0.97 (0.78, 1.22)
Current Smoker		0.58 (0.39, 0.86)
**Drug Use Disorder**		
No drug abuse or dependence		1.00 (Reference)
Drug abuse or dependence during lifetime		0.79 (0.47, 1.35)
Missing Data		1.02 (0.67, 1.55)
**Alcohol Use Disorder**		
No alcohol abuse or dependence		1.00 (Reference)
Alcohol abuse or dependence during lifetime		1.32 (0.99, 1.76)
Missing Data		**2.65 (1.62, 4.34)**
**Spirituality Level**		
Very important		1.00 (Reference)
Somewhat or not very important		1.01 (0.74, 1.37)
Not important at all		1.14 (0.86, 1.51)
Missing data		1.75 (0.71, 4.32)
**Can Count on People You Know to Deal With the Biggest Stress**		
Strongly agree		1.00 (Reference)
Neither agree nor disagree		0.71 (0.48, 1.04)
Disagree/Strongly Disagree		0.91 (0.62, 1.35)
Missing data		0.58 (0.36, 0.93)
**Frequency of Warm and Trusting Relationship With others During the Past Month**		
Daily		1.00 (Reference)
Almost daily		0.91 (0.72, 1.15)
2-3 times a week including never		0.98 (0.72, 1.34)
Missing data		1.32 (0.73, 2.39)
**Pain Preventing Activities**		
No pain or no activities prevented by pain		1.00 (Reference)
Pain prevents few/some/most activities		1.28 (1.00, 1.64)
Missing data		1.15 (0.45, 2.92)
**Chronic Conditions (not cancer)**		
No chronic condition		1.00 (Reference)
1 chronic condition		0.93 (0.72, 1,21)
2 chronic conditions		1.19 (0.90, 1.58)
3 or more chronic conditions		0.98 (0.70, 1.36)
Missing data		1.36 (0.80, 2,30)
**Nagelkerke Pseudo R² values** **P-value of the Block**	0.02 P < 0.001	0.06 P < .0.001

2022 Canadian Mental Health and Access to Care Survey Data (MHACS)

Bold indicates statistical significance with p < 0.05

An unexpected finding in our fully adjusted model was that White respondents had higher odds of reporting a cancer diagnosis compared to those identifying as a visible minority. This pattern contrasts with some prior work suggesting that racialized populations may experience greater cumulative adversity and stress exposure across the life course. Several explanations are possible. Visible minority groups in Canada may under-report cancer diagnoses due to barriers in healthcare access, lower screening participation, or cultural differences in disclosure. It is also possible that older White Canadians in this sample had higher historical exposure to well-established cancer risk factors, such as smoking or occupational hazards, which may contribute to this pattern. Furthermore, the majority of visible minority older Canadians are immigrants. There is a well-established healthy immigrant effect among immigrants to Canada, which is in keeping with the current findings.

We also observed significant associations for several “missing” categories (e.g., race/ethnicity, alcohol abuse). These should be interpreted with caution, as they likely reflect systematic patterns of non-response rather than true underlying relationships with cancer. Missingness in national surveys is rarely random; individuals who skip sensitive items may differ in health status, trust in institutions, or comfort disclosing personal information. As such, the elevated odds among respondents with missing data likely indicate measurement or reporting error rather than meaningful associations. This highlights the importance of cautious interpretation when working with self-reported survey data and underscores the need for improved measurement of key constructs in future research.

Prior research has emphasized smoking, obesity, and physical inactivity as key behavioral pathways linking ACEs to long-term health outcomes, including cancer [41,42]. However, the present study found that adjusting for these factors did not significantly weaken the relationship between CSVC and cancer risk, suggesting that association between ACEs and problematic health behaviors do not fully account for the observed association.

Inclusion of education level and household income, which are well-documented protective factors against cancer [43,44], did not substantially attenuate the association between CSVC and cancer. While childhood adversity is strongly associated with lower adult SES, the persistence of the CSVC–cancer link, even after accounting for these factors, suggests that socioeconomic disadvantage does not fully mediate this relationship. As was discussed above, one possible explanation is that early-life sexual violence involving coercion may exert direct biological and psychological effects on long-term health, independent of socioeconomic conditions [45]. Additionally, individuals with a history of CSA may develop maladaptive coping mechanisms, such as substance use [46], and disordered eating [47], that can contribute to cancer risk through behavioral pathways. We were able to control for substance use disorders in our logistic regression analyses, but not disordered eating.

This study also statistically adjusted for psychosocial variables, including social support and spirituality. However, the association between CSVC and cancer risk remained virtually unchanged, reinforcing the idea that early-life trauma may exert lasting biological effects that persist even in the presence of protective psychosocial resources. These findings highlight the need for future research to examine stress-related epigenetic changes, chronic inflammation, and other physiological pathways that may underlie the link between CSA and cancer in older adulthood.

### Implications for research and practice

The findings of this study emphasize the association between later-life health conditions and early-life trauma, particularly the independent association between CSVC and increased cancer risk in older adulthood. Given that this relationship persisted even after adjusting for SES, mental health conditions, health-risk behaviors, and social support, it is crucial for future research to further explore the biopsychosocial pathways linking childhood adversity to cancer in older adulthood [48].

Our findings, alongside those of Hovdestad et al. [12], highlight the importance of incorporating trauma history into preventative cancer care. Both studies found strong associations between CSVC and cancer risk, reinforcing the need for trauma-informed outreach and interventions to promote equitable access to early detection and support services [49]. Emerging research on trauma-exposed populations suggests that psychosocial barriers—such as those associated with serious mental illness and adverse childhood experiences—may inhibit engagement in cancer screening, with studies showing lower uptake of breast and cervical screening among women with serious mental illness [50] and complex, ACE-specific associations with screening behaviors [51]. Future research should explore these barriers further, as well as potential biological markers—such as inflammation and dysregulated cortisol—to inform integrated, multidisciplinary approaches to cancer survivorship care.

It has been proposed that healthcare providers should implement standardized screening protocols for childhood trauma in routine clinical practice to better identify individuals at risk for long-term health complications, including chronic disease and mental illness [52]. While our study focused on physical health outcomes, the link between CSA and psychological distress—particularly depression and post-traumatic stress disorder—has been well-established in longitudinal studies [53]. As depression has also been identified as a potential risk factor for cancer [54], future studies should consider including it as a covariate to better elucidate the pathways through which early-life trauma may influence later cancer risk. Incorporating trauma screening into cancer care could also support more holistic assessment and intervention efforts, especially when paired with access to evidence-based mental health supports. While the evidence linking ACEs and cancer is growing, findings across studies are not always consistent—likely due to methodological differences in how ACEs are measured, the types and timing of outcomes assessed, and whether studies adjust for specific psychosocial and behavioral mediators, such as chronic stress [55], psychological distress [56], and health behaviors like smoking, alcohol consumption, and obesity [57].

Furthermore, when older adults are diagnosed with cancer, incorporating evidence-based mental health interventions, such as Cognitive Behavioral Therapy (CBT) [58], or Emotion-Focused Therapy [59] may play a critical role in comprehensively addressing psychological distress. Although no studies have yet demonstrated that psychological interventions such as CBT directly reduce cancer incidence, emerging evidence suggests that these interventions can modulate systemic inflammation and stress-related biological pathways and improve health behaviors that are relevant to cancer risk and survivorship [60]. Furthermore, Managing Cancer and Living Meaningfully (CALM) therapy is a psychotherapeutic intervention designed to support patients with advanced cancer by providing emotional support, alleviating distress, and helping them navigate the complexities of living with a serious illness [61]. CALM therapy has been widely studied in palliative care, demonstrating its effectiveness in reducing existential distress and improving quality of life for individuals with advanced cancer. Given these benefits, there is growing interest in expanding CALM beyond palliative care into primary healthcare settings across Canada to enhance psychosocial support for cancer patients. Its adoption has also gained international traction, with successful implementations in the Netherlands [62], Japan [63], and Italy [64], underlining its global potential as a standard component of oncology care.

### Limitations

There are several important limitations to this research that must be considered when interpreting the results. This study examines the associations between childhood adversities—specifically CPA, CSA (unwanted touch/fondling and CSVC), and PDV—and cancer risk in older adulthood but was constrained by the use of secondary data. Although this study adjusts for several key covariates—including SES, social support, and substance use —residual confounding remains a concern. For example, malignancies involving inflammatory or immune-related pathways may be more strongly linked to early-life trauma. Investigating these relationships by cancer type would provide more precise insights into potential associative mechanisms of risk.

The MHACS data did not include several factors that have been implicated in cancer risk including body mass index (BMI) [65], information on the duration of the childhood abuse nor the perpetrator [66], genetic predisposition [67], early-life health conditions [68], poverty in childhood [69], or other childhood stressors, such as neglect, exposure to community violence, or peer victimization, all of which could contribute to long-term health vulnerabilities [5,15,70]. Future research would benefit from inclusion of anthropometric data to examine whether BMI plays a mediating role in the CSA-cancer link. Another limitation of this study is that the MHACS Public Use Dataset provides only a single aggregated age category for respondents aged 65 and older. It would be preferable in future research to be able to take into account the well-established relationship between age within 10 year cohorts and cancer risk.

Another limitation is that the study does not differentiate between cancer types. Given that cancers have distinct etiologies and risk factors, previous research suggests associations between childhood adversity and cancer may vary depending on cancer subtype [8–10]. The cross-sectional design also prohibits the determination of causality between ACEs and cancer risk, as unmeasured factors may contribute to both. Additionally, reliance on self-reported data introduces the potential for recall bias and misclassification, particularly with retrospective reporting of trauma, which may be influenced by memory limitations, stigma, or reinterpretation over time [71]. Furthermore, the cancer diagnoses in the MHACS were based on self-reports rather than medical records, potentially leading to misclassification. However, while prior research suggests self-reports of cancer diagnoses are generally accurate in large-scale epidemiological surveys [72], correctly identifying approximately 73% of true cases and accurately ruling out 96% of non-cases [73,74], they are not validated by chart review in the current study and should be in future research.

Selection bias may further affect the findings, as the MHACS is a community-based survey and does not include individuals who are hospitalized or institutionalized. Hospitalized individuals may have higher rates of both trauma exposure and serious illness, which could have resulted in an underestimation of the association between childhood adversities and cancer. Additionally, national differences in healthcare access and sociocultural factors across populations may restrict the generalizability of findings beyond Canada. Furthermore, the response rate in the MHACS was 30%, which is less than ideal. This may have led to bias in the conclusions drawn.

Despite these limitations, the current study provides a Canadian population-based study of older adults. We found that the odds of cancer among older adult survivors of CSVC were double that of their peers without a history of CSA. This association persisted even after controlling for multiple demographic, socioeconomic, behavioral, and psychosocial factors. Future longitudinal research using chart-reviews of cancer diagnoses is needed to clarify the specific biopsychosocial mechanisms underlying the childhood adversity-cancer’s relationship, incorporating factors such as chronic inflammation, immune dysregulation, and epigenetic changes that were not assessed in this study. Expanding research to explore these pathways may inform targeted prevention and intervention strategies aimed at improving the long-term health of child abuse survivors.
